# The relationship between different bispectral index and the occurrence of dreams in elective surgery under general anesthesia: protocol for a randomized controlled clinical trial

**DOI:** 10.1186/s13063-023-07222-2

**Published:** 2023-03-20

**Authors:** Yufei Zhang, Bijia Song, Junchao Zhu

**Affiliations:** Department of Anesthesiology, Shengjing Hospital of China Medical University, Shenyang, Liaoning People’s Republic of China

**Keywords:** Dreaming, Preoperative anxiety, Propofol, Satisfaction

## Abstract

**Introduction:**

Dreaming reported after anesthesia remains a poorly understood phenomenon. At present, there is a hypothesis that dreaming occurs intraoperatively and is related to light or inadequate anesthesia; thus, in order to further verify the hypothesis, we choose elective surgery under general anesthesia to observe whether the generation of dreams is related to the dose of general anesthetics maintenance.

**Methods and analysis:**

This randomized, double-blind controlled trial to observe whether the generation of dreams is related to the dose of general anesthetics maintenance in the elective surgery under general anesthesia. A total of 124 participants will be randomly allocated to a low bispectral index or high bispectral index group at a ratio of 1:1. The Hospital Anxiety and Depression Scale (HADS) is used to assess the anxiety and depression status of participants during the perioperative period. Ramsay score is used to assess patients’ sedation level after surgery in the PACU. Modified Brice questionnaire and awareness classification are used to assess whether patients experienced dreaming during the surgery.

**Ethics and dissemination:**

This randomized, double-blind controlled trial received prospective ethics committee approval at the Human Research Ethical Committee of Shengjing Hospital, Shenyang, Liaoning Province, China (Institutional Review Board registration number 2021PS664K), and was compliant with the Declaration of Helsinki. Written informed consent was obtained from all subjects participating in the trial.

## Introduction

Dreaming is a familiar and mysterious mode of cognitive function, and we involuntarily return to this mode every night. Dreaming during sleep is defined as “any type of cognitive activity that occurs during sleep” and is “a subjective experience that can only be obtained through the dreamer's memories after waking up.” Despite more than a century of scientific exploration, dreams continue to arouse the interest of sleep scientists, but they are still not fully understood [1, 2]. Moreover, its rigorous scientific exploration is a recent development, dating back to the discovery of rapid eye movement (REM) sleep in the 1950s. When this stage of sleep was first described in humans, researchers quickly noticed that people who awakened from REM sleep often reported dreaming (in 74% of cases, only 17% of non-REM [NREM] sleep). Therefore, dreaming is equivalent to rapid eye movement sleep, and this concept seems to be consistent with the electrophysiological characteristics of this sleep stage: closing the eyeballs under the eyelids, as if the sleeper is watching an animated scene [3, 4]. General anesthesia causes a drug-induced state of unconsciousness and is a non-physiological process that is similar to natural sleep. Its purpose is to create a state of sensory deprivation wherein patients are unresponsive to stimuli and thus leads to explicit amnesia [5]. Dreaming is also a common, long-lasting, and fascinating part of the anesthesia experience, but its cause and timing are still elusive. Patients usually report that they dreamed during anesthesia, but the actual time of dreaming during anesthesia is unknown. Dreaming during anesthesia can be defined as “any experience (excluding awareness) that a patient is able to recall and which he or she thinks occurred between induction of anaesthesia and the first moment of consciousness after anaesthesia” [6]. Patients receiving propofol for general anesthesia often report a higher incidence of dreaming compared with patients maintained with volatile anesthetics [7]. One explanation is that propofol and volatile anesthetics have different pharmacological effects in the central nervous system [8, 9]. Another explanation is that propofol can wake up from anesthesia faster than the volatile anesthetics, allowing patients to report their dreams before they are forgotten [10]. Why is the investigation of dreams during anesthesia important? Dreaming is one of the most common side effects of anesthesia, but it is still puzzling and requires explanation [7, 11]. Dreaming can sometimes make patients feel distressed and may reduce satisfaction with care [12]. Some patients who report dreaming worry that their anesthetic is insufficient; their experience is actually consciousness. At present, there is a hypothesis that dreaming occurs intraoperatively and is related to light or inadequate anesthesia; thus, in order to further verify the hypothesis, we choose elective surgery under general anesthesia to observe whether the generation of dreams is related to the dose of general anesthetics maintenance.

## Methods

### Participants and recruitment {10, 15}

**Fig. 1:**
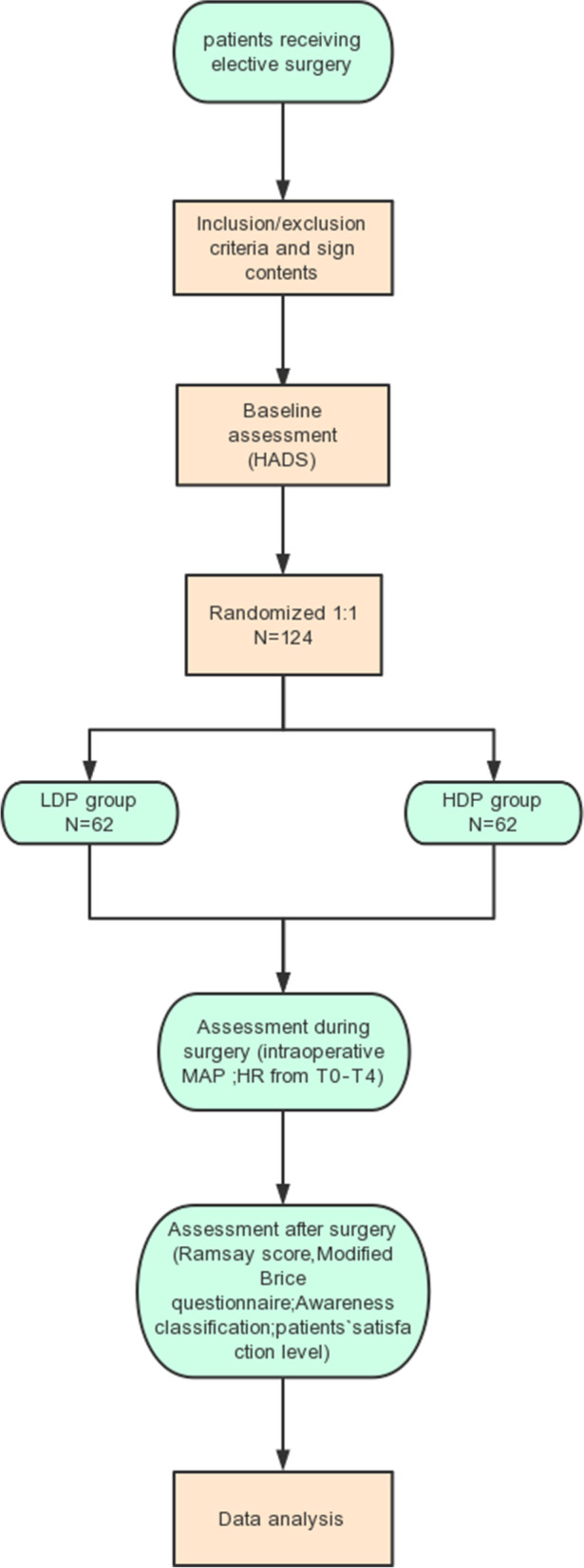
Study flowchart

With written informed consent, patients, aged 18–60 years and of American Society of Anesthesiologists’ physical status I–II, presenting for elective surgery during relaxant general anesthesia, were recruited. Exclusion criteria included (1) inadequate Chinese comprehension due to a language barrier, cognitive deficit, or intellectual disability; (2) diagnosis of a psychotic disorder, major affective disorder, or major drug dependence disorder; and (3) planned postoperative ventilation or anticipation of unavailability for postoperative interviews. A diagram of the trial design is provided in Fig. 1.

### Randomization, study intervention, and blinding {16c, 17a}

All patients were randomly divided into either the low bispectral index (LBIS) group (*n* = 62) or the high bispectral index (HBIS) group (*n* = 62). Randomization was conducted using a random-permuted block randomization algorithm. The randomization sequence was kept in sealed envelopes. On the day of surgery, the investigator opened the envelope and prepared the study drug according to the allocated group and delivered it to the operating room wrapped with opaque paper. The result of the group allocation was not opened until the time of data analysis. In all cases, anesthesia was conducted by the same anesthesiologist and outcome assessments were carried out by another anesthesiologist, and both were blinded to the group allocation. In the LBIS group, BIS was maintained between 40 and 50 during the operation, and in the HBIS group, BIS was maintained between 50 and 60 for maintenance. The patients, attending anesthesiologists, surgeons, and data collectors were all blinded to patient group assignment.

### Standardized anesthesia

All patients were instructed not to eat for 8 h or to drink for 4 h prior to procedures, and no preoperative drugs were administered. Routine monitoring devices were set up to monitor electrocardiogram, heart rate (HR), noninvasive blood pressure (BP), oxygen saturation (SpO_2_), respiratory rate (RR), and partial pressure of end-tidal carbon dioxide (PetCO_2_). O_2_ at 2 L/min was applied through a nasal catheter, and intravenous access was opened. General anesthesia was induced with propofol (2 mg/kg), sufentanil (0.2 μg/kg), and cisatracurium (0.15 mg/kg). After adequate jaw relaxation was attained, tracheal intubation was performed, and each patient was mechanically ventilated with a tidal volume and ventilation rate adjusted to maintain the pressure of end-tidal carbon dioxide (ETCO2) at 35–45 mmHg. The anesthesiologist adjusted the intravenous speed of remifentanil and propofol according to hemodynamic parameters and BIS (BIS monitor; Aspect Medical System, Newton, MA). In the LBIS group, BIS was maintained between 40 and 50 during the operation, and in the HBIS group, BIS was maintained between 50 and 60 for maintenance. Both groups received 0.15–0.2 μg/kg/min for remifentanil and together with inhaled 50% air and 50% oxygen (O_2_) at a fresh gas flow rate of 2 L/min. Cisatracurium (0.05 mg/kg) was intermittently used for muscle relaxation. Ten minutes before the end of the surgery, stop all anesthetic infusions, and after extubating, the patients were transferred to the PACU for continuous monitoring.

### Observation indicators {12}

#### General data

**Fig. 2:**
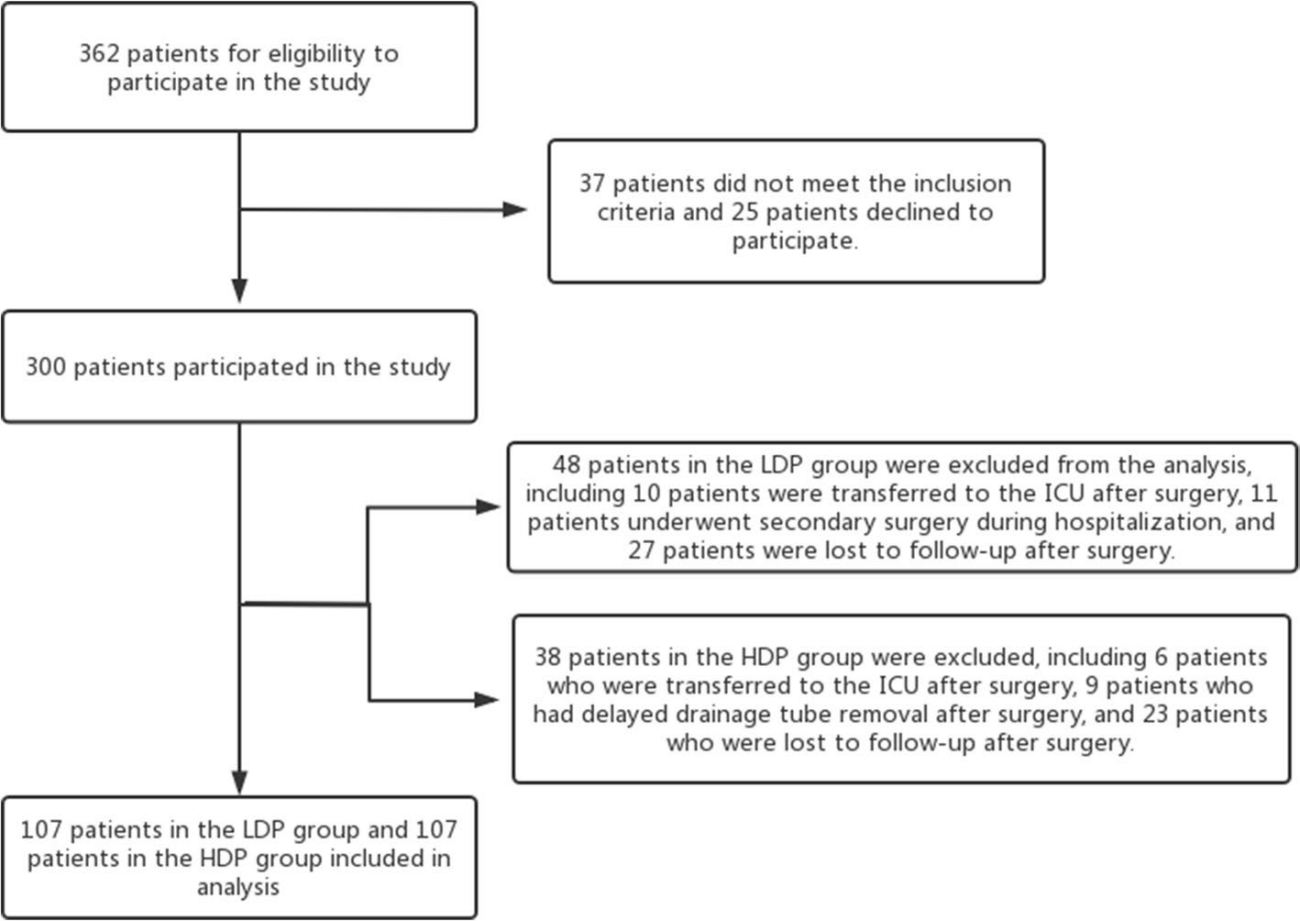
Patient inclusion and exclusion algorithm

The following patient data were collected from the anesthetic records: age, gender, height, weight, body mass index (BMI), American Society of Anesthesiologists physical status (ASA), duration of falling asleep, duration of surgery, duration of anesthesia, duration of recovery to consciousness (time between withdrawal of drugs and orientation of patients to time, place, and person), and drugs used for anesthetic management. The Hospital Anxiety and Depression Scale (HADS) is a self-report questionnaire used to assess the anxiety and depression status of participants during the perioperative period (Fig. 2). We used the Japanese version of HADS in this study. HADS consists of two subscales: anxiety (HADS-A) and depression (HADS-D). Each score consists of 7 questions, ranging from 0 to 3 (each score is 0–21), depending on the probability of the psychological problem described. Patients with a score of 11 or higher can be classified as possibly significant [13, 14]. We evaluated HADS score of patients the day before surgery.

#### Intraoperative data

Mean arterial pressure (MAP) and HR of each patient were recorded at 5 min after entering the operation room (T0), after anesthesia induces to fall asleep (T1), at the end of the operation (T2), recovery from anesthesia (T3), and exiting from the operation room (T4). Record the total amount of propofol used during surgery.

#### Postoperative data

Record the Ramsay score during orientation recovery (Ramsay sedation scale: 1 = patient anxious, agitated or restless or both; 2 = patient cooperative, oriented, tranquil, and alert; 3 = patient responds to commands; 4 = asleep, but with brisk response to light glabellar tap or loud auditory stimulus; 5 = asleep, sluggish response to light glabellar tap or loud auditory stimulus; 6 = asleep, shows no response to light glabellar tap or loud auditory stimulus) [15]. Record the patients’ satisfaction level in the PACU: fairly dissatisfied, medium satisfied, most satisfied, very satisfied.

#### Time and method of dreaming investigation

After emergence from sedation and orientation recovery, a modified Brice questionnaire was immediately used to evaluate the incidence of dreaming. Patients who reported dreaming were required to complete an awareness classification regarding the awareness level of their dreams.

#### Modified Brice questionnaire [16]

1. What was the last thing you remember before going to sleep?
2. What was the first thing you remember from when you woke up?
3. Can you recall anything between?
4. Did you have any dreams while under anesthesia?
5. How did you feel about your dream: pleasant, indifferent, or unpleasant?

#### ***Awareness classification ***[17] 

1. No awareness: no reported awareness or a vague description, or what had been reported had a high probability of occurring in the immediate pre- or postoperative period; i.e., music, people talking, dressing application
2. Dreaming, possibly associated with awareness
3. Possible awareness: patient unable to recall any event definitely indicative of awareness
4. Awareness: recalled event was confirmed by attending personnel, or the investigators were convinced that the memory was real, but no confirmation could be obtained

#### Strategies to improve adherence to interventions {11c}

The investigators will explain the treatment intervention in detail and supervise the compliance of the intervention throughout the entire procedure (during maintenance of anesthesia). Periodic monitoring will be conducted by a blinded investigators, for the recording of postoperative parameters and to ensure adherence to interventions.

#### Relevant concomitant care permitted or prohibited during the trial {11d}

Usual induction of anesthesia is permitted for both intervention and control groups. Adverse effects such as hypotension, bradycardia, nausea and vomiting, and hypoxemia were also treated accordingly. Participation in additional research trials is prohibited during the trial.

#### Safety assessment {22}

The anesthesiologist adjusted the intravenous speed of remifentanil and propofol according to hemodynamic parameters and BIS (BIS monitor; Aspect Medical System, Newton, MA). The BIS was maintained between 40 and 60 during the operation, which avoids the incidence of intraoperative awareness in patients in both groups.

During the study period, the adverse event record form is completed to record the duration, occurrence time, severity, measures, and outcome of the adverse event. The researchers will report it to the person in charge of the trial, the sponsoring unit, and the medical ethics committee in time. The ethics committee have the right to make the final decision if termination is needed.

#### Provisions for post-trial care {30}

There is no anticipated harm and compensation for trial participation.

#### Procedure for unblinding if needed {17b}

At the end of the study, the original data and results will be submitted to the scientific research management committee, and they will be disclosed to the public after the results are published.

#### Plans to promote participant retention and complete follow-up {18b}

Full communication before surgery, in order to dispel the patient’s fear; explanation of the specific situation to establish trust between doctors and patients; and obtaining of informed consent were performed. A verbal explanation of the written consent will be provided by the research member, and any questions regarding the study will be answered. Each participant will have sufficient time to decide whether to participate in this study. If patients are willing to participate, written consent will be obtained.

#### Data management {19, 27}

All personal information about the participants will be collected and stored in a secure cabinet by the lead investigators, throughout the duration of the study, to guarantee confidentiality. Only the lead investigator will have access to the files corresponding to the personal data of the participants.

#### Protocol amendments {25}

The study plan of this project has been registered with approval number 2021PS664K. If any changes to the protocol are required, we will first notify the sponsor and funder; then, the principal investigator (PI) will notify the center, and a copy of the revised protocol will be sent to the PI for addition to the investigator site file. Any deviation from the agreement will be fully documented using the violation reporting form. The PI for this study will update the protocol in the Clinical Trials Registry.

### Statistical analysis

#### Sample size

In this trial, according to the previous study [18], the total dose of propofol in the dream group was 337 ± 161 mg and by 259 ± 145 mg in the no dream group. Using a *t* test of two independent samples in PASS for calculation, the withdrawal rate is 20% to ensure that the results are statistically different (*α* = 0.05, 1-*β* = 0.8); therefore, each group requires 56 subjects. To improve the reliability of the trial, we will recruit a total of 124 patients, with 62 in each group.

The SPSS 20.0 statistical software (SPSS, Inc., Chicago, IL, USA) and GraphPad Prism 6.0 software were used for data analysis. Continuous data were tested for normality. Normally distributed data were summarized using mean and standard deviation and were compared using unpaired two-tailed *t*-tests. Skewed data were summarized using.median and range and were compared using Wilcoxon rank-sum tests. Categorical data were summarized using number (%) and were compared using *Χ*^2^ test or Fisher’sexact tests. Logistic regression was used to determine predictors of dreaming and correlation with dream quality. Categorical variables were created from continuous variables in applicable cases. A *P* value of less than 0.05 was considered statistically significant.

#### Methods in analysis to handle protocol non-adherence and any statistical methods to handle missing data {20c}

All researchers will be trained referring to the same training protocol. Missing intraoperative data, if any, will be obtained from the electronic hospital files. Postoperative evaluation at specified time points is mandatory, and missing postoperative data are not to be anticipated. Analyses of all outcomes will be performed according to the intention-to-treat principle, and once enrolled, all participants will be analyzed, regardless of the findings.

## Discussion

Dreams are a remarkable experiment in psychology and neuroscience, conducted every night in every sleeping person. They show that the human brain, disconnected from the environment, can generate an entire world of conscious experiences by itself. The occurrence of dreams is also one of the common phenomena of anesthesia and sedation. The occurrence of dreams during general anesthesia has been reported in the literature. The dream after anesthesia refers to the patient’s experience after the induction of anesthesia to the beginning of the waking phase. Domestic scholars have conducted research on the effects of short-town intravenous anesthesia and specific anesthetics on dreams of adult receiving surgery, which found that the incidence of adult dreams is about 18% ~ 49.7% [19, 20]. Among them, different anesthesia drugs, operation site, operation time, and so on are all the influential factors leading to the occurrence of dreams. Previous hypothesis also thought that dreaming occurs intraoperatively and is related to light or inadequate anesthesia [21, 22]. In order to verify this previous hypothesis, we designed a randomized, observer- and patient-blinded, controlled clinical trial, which compared the effects of different maintenance doses of propofol on patients’ dreams and analyzed the related factors affecting dreams. A recent study has shown that depression and anxiety assessed by Beck anxiety/depression inventory can predict a negative dream during natural sleep [23]. Similarly, we assessed preoperative anxiety level to see if patients with different preoperative anxiety states were associated with different dreams generated during anesthesia. In our trial, the subjective outcomes including HADS, Ramsay scale, modified Brice questionnaire, and awareness classification will be assessed. In this way, the effect of different dose of propofol maintained during the surgery on the incidence of dreaming of patients will be evaluated from multiple perspectives.

Our study has some limitations. First, since all the patients were hospitalized, we only scored the preoperative anxiety during the perioperative period but did not evaluate the daily long-term psychological base state and the degree of dreaming under natural sleep. Second, the study was part of a single-center survey, multicenter survey still need to be studied.

## Conclusion

In conclusion, the results of our trial are expected to demonstrate the proportion of patients in the HBIS group who had dreams during the surgery vs that of patients in the LBIS group and whether there are patient-reported awareness during anesthesia. Through all the patients who reported dreaming during the surgery, we are aimed to explore the proportion of those who reported having pleasant dreams in the LBIS group vs that of patients in the HBIS group, to further compare whether there is a difference in the dosage of propofol between the two groups, and to analyze the factors associated with different postoperative dream generation. In summary, according to the study results, we hope to provide a basis for good perioperative experience and better postoperative recovery of patients in clinical practice.

## Trial status

The participant recruitment was finished and data collection are in progress. Protocol version number is 1.0 and date: 2021.11; the date recruitment began on November 30, 2021, and an estimate of the date would be April 30, 2022, when recruitment will be completed.

## Data Availability

N/A.

## References

[1] Siclari F, Baird B, Perogamvros L (2017). The neural correlates of dreaming. Nat Neurosci.

[2] Flaskerud JH (2018). Dreaming. Issues Ment Health Nurs.

[3] Picard-Deland C, Nielsen T, Carr M (2021). Dreaming of the sleep lab. PLoS ONE.

[4] Leclair-Visonneau L, Oudiette D, Gaymard B, Leu-Semenescu S, Arnulf I (2010). Do the eyes scan dream images during rapid eye movement sleep? Evidence from the rapid eye movement sleep behaviour disorder model. Brain.

[5] Selvadurai S, Maynes JT, McDonnell C (2018). Evaluating the effects of general anesthesia on sleep in children undergoing elective surgery: an observational case-control study. Sleep.

[6] Matus H, Kvolik S, Rakipovic A, Borzan V (2021). Bispectral index monitoring and observer rating scale correlate with dreaming during propofol anesthesia for gastrointestinal endoscopies. Medicina (Kaunas).

[7] Hellwagner K, Holzer A, Gustorff B, Schroegendorfer K, Greher M, Weindlmayr-Goettel M, Saletu B, Lackner F (2003). Recollection of dreams after short general anaesthesia: influence on patient anxiety and satisfaction. Eur J Anaesthesiol.

[8] Grasshoff C, Drexler B, Rudolph U, Antkowiak B (2006). Anaesthetic drugs: linking molecular actions to clinical effects. Curr Pharm Des.

[9] Rudolph U, Antkowiak B (2004). Molecular and neuronal substrates for general anaesthetics. Nat Rev Neurosci.

[10] Gupta A, Stierer T, Zuckerman R, Sakima N, Parker S, Fleisher L (2004). Comparison of recovery profifile after ambulatory anesthesia with propofol, isoflflurane, sevoflflurane and desflflurane: a systematic review. Anesth Analg.

[11] Mashour GA (2011). Dreaming during anesthesia and sedation. Anesth Analg.

[12] Leslie K, Myles P, Forbes A, Chan M, Swallow S, Short T (2005). Dreaming during anaesthesia in patients at high risk of awareness. Anaesthesia.

[13] de Almeida Macêdo E, Appenzeller S, Lavras Costallat LT. Assessment of the Hospital Anxiety and Depression Scale (HADS) performance for the diagnosis of anxiety in patients with systemic lupus erythematosus. Rheumatol Int. 2017;37(12):1999–2004Annunziata MA, Muzzatti B, Bidoli E, et al. Hospital Anxiety and Depression Scale (HADS) accuracy in cancer patients. Support Care Cancer. 2020;28(8):3921–3926. . 10.1007/s00296-017-3819-x 28940018

[14] Lozano-Díaz D, Valdivielso Serna A, Garrido Palomo R, Arias-Arias Á, Tárraga López PJ, Martínez GA (2021). Validation of the Ramsay scale for invasive procedures under deep sedation in pediatrics. Paediatr Anaesth.

[15] Moonesinghe SR, Walker EM, Bell M, SNAP-1 Investigator Group. Design and methodology of SNAP-1: A Sprint National Anaesthesia Project to measure patient reported outcome after anaesthesia. Perioper Med (Lond), 2015;4:4. 10.1186/s13741-015-0011-2 PMC4422533 25949810

[16] Sebel PS, Bowdle TA, Ghoneim MM (2004). The incidence of awareness during anesthesia: a multicenter United States study. Anesth Analg.

[17] Eer AS, Padmanabhan U, Leslie K (2009). Propofol dose and incidence of dreaming during sedation. Eur J Anaesthesiol.

[18] Leslie K, Skrzypek H (2007). Dreaming during anaesthesia in adult patients. Best Pract Res Clin Anaesthesiol.

[19] Strickland RA, Butterworth JF (2007). Sexual dreaming during anesthesia: early case histories (1849–1888) of the phenomenon. Anesthesiology.

[20] Myles P, Leslie K, McNeil J, Forbes A, Chan M (2004). A randomised controlled trial of BIS monitoring to prevent awareness during anaesthesia: The B-Aware Trial. Lancet.

[21] Huang G, Davidson A, Stargatt R (2005). Dreaming during anaesthesia in children: Incidence, nature and associations. Anaesthesia.

[22] Komasi S, Soroush A, Khazaie H, Zakiei A, Saeidi M (2018). Dreams content and emotional load in cardiac rehabilitation patients and their relation to anxiety and depression. Ann Card Anaesth.

[23] Palagini L, Rosenlicht N. Sleep, dreaming, and mental health: a review of historical and neurobiological perspectives. Sleep Med Rev. 2011;15(3):179–86. 10.1016/j.smrv.2010.07.003 20850358

